# Machine learning algorithms to uncover risk factors of breast cancer: insights from a large case-control study

**DOI:** 10.3389/fonc.2023.1276232

**Published:** 2024-02-15

**Authors:** Mostafa Dianati-Nasab, Khodakaram Salimifard, Reza Mohammadi, Sara Saadatmand, Mohammad Fararouei, Kosar S. Hosseini, Behshid Jiavid-Sharifi, Thierry Chaussalet, Samira Dehdar

**Affiliations:** ^1^ School of Medical and Life Sciences, Sunway University, Sunway City, Malaysia; ^2^ Department of Epidemiology, School of Public Health, Shiraz University of Medical Sciences, Shiraz, Iran; ^3^ Computational Intelligence & Intelligent Optimization Research Group, Business & Economics School, Persian Gulf University, Bushehr, Iran; ^4^ Department of Operation Management, Amsterdam Business School, University of Amsterdam, Amsterdam, Netherlands; ^5^ Department of Medicine, Iran University of Medical Sciences, Tehran, Iran; ^6^ Computer Science and Engineering, University of Westminster, London, United Kingdom

**Keywords:** breast cancer, machine learning, risk factor, random forest, neural networks, bootstrap aggregating classification and regression tree, extreme gradient boosting

## Abstract

**Introduction:**

This large case-control study explored the application of machine learning models to identify risk factors for primary invasive incident breast cancer (BC) in the Iranian population. This study serves as a bridge toward improved BC prevention, early detection, and management through the identification of modifiable and unmodifiable risk factors.

**Methods:**

The dataset includes 1,009 cases and 1,009 controls, with comprehensive data on lifestyle, health-behavior, reproductive and sociodemographic factors. Different machine learning models, namely Random Forest (RF), Neural Networks (NN), Bootstrap Aggregating Classification and Regression Trees (Bagged CART), and Extreme Gradient Boosting Tree (XGBoost), were employed to analyze the data.

**Results:**

The findings highlight the significance of a chest X-ray history, deliberate weight loss, abortion history, and post-menopausal status as predictors. Factors such as second-hand smoking, lower education, menarche age (>14), occupation (employed), first delivery age (18-23), and breastfeeding duration (>42 months) were also identified as important predictors in multiple models. The RF model exhibited the highest Area Under the Curve (AUC) value of 0.9, as indicated by the Receiver Operating Characteristic (ROC) curve. Following closely was the Bagged CART model with an AUC of 0.89, while the XGBoost model achieved a slightly lower AUC of 0.78. In contrast, the NN model demonstrated the lowest AUC of 0.74. On the other hand, the RF model achieved an accuracy of 83.9% and a Kappa coefficient of 67.8% and the XGBoost, achieved a lower accuracy of 82.5% and a lower Kappa coefficient of 0.6.

**Conclusion:**

This study could be beneficial for targeted preventive measures according to the main risk factors for BC among high-risk women.

## 1 Introduction

Breast cancer (BC) stands as the foremost cause of cancer-related deaths among females and remains a significant global health concern, with over 2.3 million new cases and 685,000 deaths solely in 2020 (1, 2). BC is anticipated to experience a considerable increase in cases by 2030, driven by significant lifestyle changes, as forecasted by the World Health Organization (WHO) (3).

BC is a prevalent cancer among Iranian women, accounting for nearly a third of all cancer occurrences (4) and it has been on an upward trajectory in recent years, reaching an age-standardized rate of prevalence of 47.1 per 100,000 Iranian women in 2018 (5). BC in Iranian women typically manifests at an earlier age and follows a more aggressive clinical course in comparison to Western populations (6). The concerning attributes of BC in Iran underscore the necessity for specific prevention and treatment strategies that take the population’s lifestyle and demographic characteristics into consideration.

While extensive research has been conducted to identify BC risk factors and preventive measures (7–9) the complex and multifactorial nature of BC necessitates innovative approaches for a comprehensive analysis. In recent years, advancements in machine learning (ML) techniques have showcased a promising future across various medical fields, including cancer research.

ML is known as a branch of artificial intelligence (AI) that relies upon a diverse set of statistical, optimization, and probabilistic techniques, facilitating computers in gathering insights from previous examples and identifying subtle patterns in complex datasets (10). These techniques have demonstrated high potential in identifying relevant factors and crafting personalized prevention strategies for different types of cancer (11). Consequently, ML models possess the capability to harness extensive datasets, extract invaluable insights from intricate patterns, and facilitate the identification of risk factors that may have been disregarded through traditional statistical methodologies (12, 13).

The application of ML models in cancer research has shown promising results in improving risk prediction, prognosis estimation, and treatment selection (14). These models can integrate diverse sets of data, including clinical, genetic, lifestyle, and environmental factors, to generate accurate risk profiles for individuals. By employing the power of ML algorithms, researchers can analyze complex interactions among various risk factors and identify high-risk individuals who can benefit the most from tailored preventive interventions (15, 16).

While ML models have been successfully employed in BC research globally (14), their application in the context of Iranian population-specific risk factors and preventive measures remains limited. Like numerous other nations, Iran exhibits distinct patterns concerning the incidence rate among younger age groups, the clinical attributes of this health issue, and the social and cultural surroundings of individuals dealing with BC (17). Therefore, exploring the application of ML models to identify risk factors and preventive measures specific to the Iranian population can provide valuable insights for tailored interventions and resource allocation in terms of disease control and prevention.

By integrating data from diverse sources and leveraging advanced ML algorithms, the complex etiology of BC is further understood through the advancements made by this research. The primary objective of this study was to investigate the potential of different ML models in identifying risk factors associated with primary invasive BC to develop more personalized preventive measures within the Iranian population. We aimed at training ML models that can accurately predict BC risk factors among women.

## 2 Materials and methods

**Figure 1:**
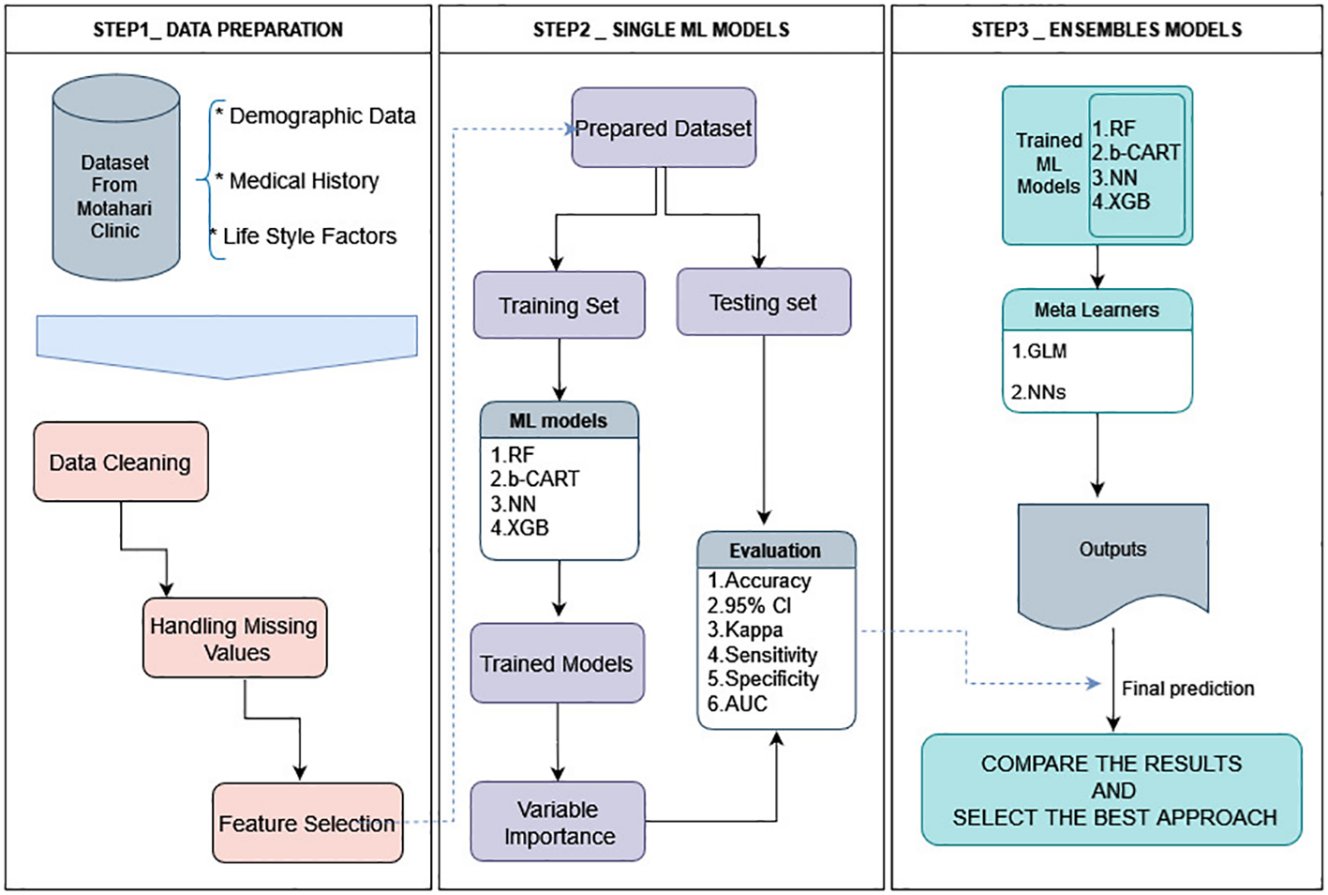
Architecture of the framework of this study. All data analysis and modeling procedures were conducted utilizing R4.2.1 programming.

The present study introduces a comprehensive framework comprising three distinct steps, as illustrated in **Figure 1**. The initial phase delineates the construction of the database and elucidates a series of meticulous operations performed to preprocess the data, ensuring its suitability for subsequent modeling endeavors. These operations encompass the integration of disparate datasets, careful data cleansing, handling of missing values, and variable selection processes for the subsequent application of ML models.

The output of this preliminary phase serves as a pivotal input for the subsequent two tiers of analysis. The second step involves the application of four diverse ML algorithms aimed at generating accurate predictions, which are subsequently evaluated using a range of statistical indices and receiver operating characteristic (ROC) curves. An ensemble approach is employed in the third and final step, where both a linear and a non-linear meta-learner algorithm forecast the anticipated outcomes.

Next, we conduct a comparative analysis between the results obtained from the second and third steps to identify the most optimal approach. In the subsequent sections, we provide a complete and detailed explanation of these processes, enabling a thorough understanding of the employed methodology. All data analyses and modeling procedures were conducted utilizing R4.2.1. programming.

### 2.1 Study population

Conducted at the Motahari Breast Clinic within Namazi Hospital, falling within the affiliation of Shiraz University of Medical Sciences in Iran, this large case-control study centered on women diagnosed with primary invasive BC. The clinic serves as the primary referral center for patients recently diagnosed with BC in the Fars province, with over 80% of these patients’ receiving treatment at this facility. The study included all eligible women with confirmed diagnosis of BC during the study period. Information about the study participants and methodology (including criteria for selecting cases and controls) has been detailed elsewhere (18).

Briefly, a total of 1,073 women were invited to participate in the study, of whom 64 were disqualified due to absent or insufficient information on histopathological reports, leaving a final sample of 1,009 cases. Written informed consent was obtained from patients who were literate, while verbal consent was obtained from illiterate patients. The study protocol was approved by the ethical committee of Shiraz University of Medical Sciences (no. 13748).

### 2.2 Case and control selection

In this case-control study, new patients with a confirmed diagnosis of BC and were admitted to the oncology and radiotherapy wards were included as incident cases. Between April 2014 and March 2017, control participants were chosen from among female attendees who were not previously diagnosed with BC and were visiting patients in different departments of the same hospital. Women in the control group were considered cancer-free if they verbally confirmed no current or history of cancer, without the need for a confirmatory examination or test. A final total of 1009 control participants were selected, frequency-matching to cases by age, using 5-year age-groups for matching (19).

### 2.3 Data collection

In a face-to-face context and within a timeframe of 2 to 8 weeks from the diagnosis of BC, interviews were carried out with the patients. Interviews took place in a private and quiet room in the hospital, facilitated by a trained female nurse. The questionnaire included questions related to education, occupation, family history of BC, smoking during adolescence and adulthood, history of oral contraceptive use, history of chest X-ray, history of benign breast disease, physical activity, body mass index (BMI), deliberate weight loss after 18 years of age, age at first delivery, total number of months of breastfeeding, history of miscarriage, menarche age, regular menstrual cycles, menopausal status, and history of type 2 diabetes. This questionnaire’s reliability has been discussed before (19).

### 2.4 Feature selection

In this research, we deliberately decided, after consulting with knowledgeable professionals in the area, to exclude the use of machine learning approaches for feature selection. This conclusion is based on recognizing the need to conduct a comprehensive examination and evaluation of all possible factors instead of just depending on automated selection techniques powered by ML algorithms.

### 2.5 Data description

**Table 1** provides insights into the distribution of the study variables among individuals without and with BC, highlighting potential associations with the condition.

**Table 1:** Statistics of BC patients and individuals without cancer.

Factors		Control (N=1009)	BC patients (N=1009)	Total (N=2018)
Education				
	Primary or illiterate	349 (35%)	348 (34%)	697 (35%)
	Intermediate	206 (20%)	179 (18%)	385 (19%)
	High school	264 (26%)	300 (30%)	564 (28%)
	Academic	190 (19%)	182 (18%)	372 (18%)
Occupation				
	Housewife	780 (77%)	779 (77%)	1559 (77%)
	Employed	229 (23%)	230 (23%)	459 (23%)
Family history of BC				
	No	867 (86%)	753 (75%)	1620 (80%)
	Second relative	54 (5%)	84 (8%)	138 (7%)
	First relative3	88 (9%)	172 (17%)	260 (13%)
Smoking				
	No	937 (93%)	860 (85%)	1797 (89%)
	Yes	72 (7%)	149 (15%)	221 (11%)
OCP use				
	Never	601 (60%)	537 (53%)	1138 (56%)
	Ever	408 (40%)	472 (47%)	880 (44%)
Chest X-ray history				
	No	317 (31%)	356 (35%)	673 (33%)
	Yes	692 (69%)	653 (65%)	1345 (67%)
History of benign breast disease				
	No	943 (93%)	869 (86%)	1812 (90%)
	Yes	66 (7%)	140 (14%)	206 (10%)
Physical activity5				
	No	799 (79%)	815 (81%)	1614 (80%)
	Yes	210 (21%)	194 (19%)	404 (20%)
BMI				
	<24.99	359 (36%)	310 (31%)	669 (33%)
	25.00 to 29.99	489 (48%)	455 (45%)	944 (47%)
	≥30.00	161 (16%)	244 (24%)	405 (20%)
Deliberate weight loss				
	No	643 (64%)	663 (66%)	1306 (65%)
	Yes	366 (36%)	346 (34%)	712 (35%)
Age at first delivery (year)				
	<18	355 (35%)	246 (24%)	601 (30%)
	18–23	284 (28%)	306 (30%)	590 (29%)
	24–30	158 (16%)	170 (17%)	328 (16%)
	≥31	131 (13%)	203 (20%)	334 (17%)
	Nulliparous	81 (8%)	84 (8%)	165 (8%)
Breastfeeding (month)				
	0–5	184 (18%)	234 (23%)	418 (21%)
	6–17	53 (5%)	86 (9%)	139 (7%)
	18–29	128 (13%)	134 (13%)	262 (13%)
	30–41	116 (11%)	108 (11%)	224 (11%)
	≥42	528 (52%)	447 (44%)	975 (48%)
History of miscarriage				
	No	694 (69%)	661 (66%)	1355 (67%)
	Yes	315 (31%)	348 (34%)	663 (33%)
Menarche age (year)				
	<12	138 (14%)	169 (17%)	307 (15%)
	12–13	431 (43%)	407 (40%)	838 (42%)
	≥14	440 (44%)	433 (43%)	873 (43%)
Regular menstruation				
	No	647 (64%)	612 (61%)	1259 (62%)
	Yes	362 (36%)	397 (39%)	759 (38%)
Menopausal status6				
	Pre-menopausal	139 (14%)	124 (12%)	263 (13%)
	Post-menopausal	868 (86%)	885 (88%)	1753 (87%)
Type 2 diabetes				
	No	942 (93%)	924 (92%)	1866 (92%)
	Yes	67 (7%)	85 (8%)	152 (8%)

The provided table presents a comprehensive overview of various factors and their distribution among the two groups. A thorough analysis of the data reveals several noteworthy observations.

In terms of education, a relatively similar distribution is observed in the two groups, with a predominant presence of individuals with primary/illiterate or intermediate education. Both groups exhibit comparable proportions in this regard.

Regarding employment, most individuals in both groups were identified as housewives, constituting approximately 77% of the total population. The remaining individuals were classified as employed, comprising around 23%. This occupational distribution is consistent across the two groups. Exploring the family history of BC reveals a substantial distinction between the groups. Among BC patients, there is a notably higher percentage (17%) of individuals with a first relative affected by BC, compared to the “control” group (9%). This disparity suggests a potential association between familial history and the incidence of BC.

Considering smoking habits, there is an appreciable difference between the groups, as the prevalence of smoking is higher among the patients (15%) compared to the controls (7%). The use of oral contraceptive pills (OCP) demonstrates a slight difference between the two groups. BC patients exhibit a slightly higher proportion (47%) of OCPs use, in contrast to the “control” group (40%).

The presence of a history of benign breast disease presents a noteworthy contrast between the two groups. Among BC patients, a greater proportion (14%) had a history of benign breast conditions, while a smaller percentage (7%) is observed in the control group. This discrepancy suggests a potential link between prior benign breast conditions and an increased susceptibility to BC.

The remaining factors in the table, including chest X-ray history, physical activity, BMI, age at first delivery, breastfeeding duration, history of miscarriage, menarche age, regular menstruation, menopausal status, and type 2 diabetes, necessitate further **s**crutiny and analysis to determine their potential implications for BC risk.

## 3 Machine learning methods

The Caret package’s grid search method (Kuhn, 2008) in R was employed to optimize hyperparameters for all algorithms (RF, NN, XGBoosting Tree, and bagged CART) within the training set. The parameter values for each applied ML algorithm are presented in **Table 1**.

### 3.1 Random forest

RF is an ensemble learning method that uses decision trees to predict classes in the case of classification or means for regression within the individual trees (20). The algorithm constructs a multitude of decision trees, and each tree is trained on a random subset of the training data and a random subset of the features. This helps to reduce overfitting by creating a diverse set of trees that are not highly correlated with each other. Overfitting is further prevented by randomly selecting a subset of features while constructing each tree. This is controlled by the hyperparameter mtry, which determines the number of variables randomly sampled at each split time. The optimal value of mtry is determined by using a grid search method (21) and was found to be 18 here with the Caret package.

### 3.2 Bagged cart

Bootstrap aggregating (bagging) is an ensemble meta-algorithm designed to enhance the stability and accuracy of ML algorithms using techniques such as classification and regression trees (CART). This involves generating multiple training sets through the process of resampling the original dataset with replacement. Subsequently, a model is trained on each of these newly created sets, leading to improved performance (22). The final prediction is then made by combining the predictions of all the generated models. Bagged CART, a variation of decision tree algorithm, employes bagging to reduce overfitting by randomly selecting a subset of features at each split to construct each tree (23). The performance of bagged CART depends on the values of hyperparameters such as the number of trees (B), the number of variables randomly sampled at each split time (mtry), and the minimum number of observations required to split an internal node (minsplit). Optimal values of these hyperparameters can be obtained through hyperparameter tuning.

The optimization process involved grid search, a method that systematically explores all possible combinations of hyperparameters within predefined ranges. The optimal values for hyperparameters were obtained by comparing the cross-validation performance of the hyperparameters mtry and minsplit control the complexity of each tree, while the hyperparameter B controls the number of trees in the ensemble. The optimal values of these hyperparameters depend on the characteristics of the dataset and the specific problem at hand. After the hyperparameter tuning was carried out, it was determined that the optimal values were as follows: mtry = [tuned value], minsplit = [tuned value], and B = [tuned value]. These tuned hyperparameter values play a crucial role in significantly enhancing the performance of Bagged CART, resulting in the creation of a ML model that is both more accurate and robust. The optimization of hyperparameters through grid search is a crucial step in developing effective ML models, and it ensures that our model can generalize well to unseen data and tackle real-world challenges effectively.

### 3.3 Neural networks

Neural Networks (NN) have captured significant attention in recent years due to their remarkable capacity to effectively address intricate challenges spanning a wide range of domains. Inspired by the structure of biological NN (24), this method employs a three-layered feedforward network. The key innovation lies in the notion of weights, which connects the hidden layers and facilitates learning between the output and input layers (25). By leveraging these weighted connections, NN excels at learning and adapting to complex patterns, making them a powerful tool in modern ML applications. One of the primary advantages of NN is their ability to learn from data without being explicitly programmed. This is achieved through a process known as backpropagation, where the network adjusts its parameters to minimize the difference between its predictions and the true outputs in the training data (26).

A multilayer perceptron (MLP) is a fully connected class of feedforward artificial neural network. To optimize the MLP, three hyperparameters were considered in this study: the number of neurons in the hidden layer, the learning rate, and the activation function. The number of neurons in the hidden layer determines the complexity of the model, with a higher number of neurons increasing model complexity (27). The learning rate governs the step size within the gradient descent optimization algorithm employed for training the network. Typically, a smaller learning rate leads to improved convergence and accuracy, optimizing the training process (28). The activation function applies a nonlinear transformation to the output of each neuron to introduce nonlinearity into the model (29).

### 3.4 Extreme gradient boosting tree

XGBoost is a popular gradient boosting algorithm that constructs an ensemble of decision trees sequentially, with each tree aiming to correct the errors of its predecessor (30). Among its many variants, XGBoost stands out as it uses decision trees as the base learner (31).

The primary objective of this study is to optimize the hyperparameters specifically for XGBoost. These hyperparameters encompass several key aspects, including the maximum depth of the trees, the learning rate (eta), the minimum child weight, the subsample ratio of columns when constructing each tree, and the gamma parameter. The parameter known as gamma, which is the focus here, holds significant importance in the context of this study. It plays a central role in establishing the minimum loss reduction required to initiate an additional partition on a leaf node, shaping the decision-making process within the algorithm (32).

The mathematical formulas for the hyperparameters are as follows:

**- Maximum depth:** The maximum depth of the decision trees, denoted as max_depth. A higher depth allows the model to capture more complex interactions but may also lead to overfitting.
**- Eta**: The learning rate, denoted as eta, controls the step size taken during the optimization process. A lower value results in slower learning but may improve generalization.
**- Minimum child weight**: The minimum sum of instance weight needed in a child, denoted as min_child_weight. It controls the minimum number of instances required in each leaf node, which helps prevent overfitting.
**- Subsample**: The subsample ratio of columns when constructing each tree, denoted as subsample. A lower value results in more conservative models.
**- Gamma**: The minimum loss reduction required to make a further partition on a leaf node, denoted as gamma. A higher value results in fewer splits and more conservative models.

Tuning these hyperparameters can lead to better performance and prevent overfitting of the model. By carefully selecting the optimal values for these parameters, XGBoost can achieve high accuracy and better generalization in real-world applications. These values are shown in **Table 2**.

**Table 2:** Parameter values of the four applied ML algorithms.

Algorithm	Parameters	Setting
**RF**	mtry (Number of variables is randomly collected to be sampled at each split time.)	18
**Bagged CART**	ensemble size	25
**Neural Network**	Hidden layer	1
	Input layer	1
	Output layer	1
	Number of neurons	5
**Extreme Gradient** **Boosting Tree**	Maximum depth	3
	Eta	0.4
	Gamma	0
	Column sample by tree	0.6
	Minimum child weight	1
	Subsample	1

## 4 Results

The results section of this study provides an in-depth analysis and presentation of the findings derived from the conducted analysis. The dataset will be subject to comprehensive examination to uncover valuable insights and observations. Meticulously exploring the dataset, characteristics, patterns, trends, and relationships are identified.

The analysis involved a comprehensive evaluation of statistical metrics and a meticulous assessment of the models’ predictive capabilities. If the circumstances warrant, the integration of these models into an ensemble framework is explored, guided by both linear and non-linear meta-learner algorithms.

### 4.1 Variable importance

In this analysis, the interaction among demographic, medical history, and lifestyle factors linked to BC risk was explored using four distinct ML models. The results provide valuable insights into the varying degrees of significance exhibited by different factors. To determine the feature importance, a well-established technique called “permutation feature importance” was utilized. This approach assesses the contribution of each predictor in the models’ decision-making process. In a specific approach, the values of each predictor were shuffled throughout the dataset, and subsequently, the resulting reduction in performance metrics of the models—such as accuracy or area under the receiver operating characteristic curve (AUC-ROC)—was assessed. The degree of decrease observed directly reflects the predictor’s significance in influencing the model’s performance. Chest X-ray history, Deliberate weight loss, abortion history, and post-menopausal status ranked among the top five predictors in all models. Moreover, secondhand smoking, education (high school), menarche age (>14), occupation (employed), first delivery age (18-23), and duration of breast-feeding (>42 months) were important in at least two models.

Conversely, certain variables, such as OCP use and physical activity, were identified as crucial only in one model, emphasizing the significance of utilizing multiple models when predicting BC risk. The rankings of particular predictors between models also varied with education (high school) and OCP use demonstrating divergent results.

The variable importance analysis emphasizes the importance of selecting an appropriate ML model and utilizing multiple models to ensure the robustness and generalizability of the predictive model. These findings suggest that a combination of demographic, medical history, and lifestyle factors should be considered when assessing BC risk. This information can be leveraged to develop targeted interventions to prevent and manage BC, contributing to the ongoing efforts to improve the accuracy and effectiveness of BC risk prediction models. These algorithms will be employed to generate predictions, and their performance will be rigorously evaluated using statistical indices and receiver operating characteristic (ROC) curves.

**Table 3** displays the importance of variables within the ML models.

**Table 3:** Variable importance.

Feature	RF	Bagged CART	XGBoost	NN
Chest X-ray history	99.78	91.46	63.47	60.05
Deliberate weight loss	98.07	99.66	86.93	–
Abortion history	97.90	93.78	–	38.48
Post-menopausal	96.36	92.52	87.60	30.44
Secondhand smoking	95.57	98.06	74.22	–
OCP use	95.20	63.15	100	100
Education	95.02	80.43	78.97	48.51
BMI (25-29.99)	94.68	91.90	55.42	28.71
Physical activity	86.02	90.99	81.18	47.69
Menarche age (12-13)	79.99	–	–	–
Menarche age (>14)	79.85	88.07	68.73	46.12
Occupation (employed)	77.30	85.52	58.59	–
First delivery Age (18-23)	74.56	73.33	55.43	–
Breast feeding duration (>42 months)	71.24	81.37	73.25	32.18
Education (intermediate)	65.55	65.44	–	–
BMI (>30)	64.22	–	67.60	35.20
Education (academic)	59.41	61.43	76.44	48.51
Smoking	55.49	–	61.22	46.64
First delivery age (24-30)	–	57.02	33.89	33.91
History of benign breast disease	–	–	79.21	41.78
Hysterectomy	–	–	–	61.98
Family history of BC (first relative)	–	–	54.64	54.64
Breast feeding duration (6-17 months)	–	–	–	91.30
Regular menstruation	–	–	59.50	–
Having diabetes	–	–	–	27.47
BMI (>24.99)	–	–	28.71	–

### 4.2 Prediction models

In this study, the prediction of outcomes using a dataset was facilitated through the utilization of four distinct machine learning algorithms. To establish a well-balanced representation, the dataset underwent a randomized partition into training and testing sets, maintaining an 80:20 ratio. Specifically, the training set, encompassing 80% of the data, was employed for model training purposes, while the remaining 20% constituted the test set, serving as the evaluative benchmark to assess model performance.

To ensure the validity and reliability of our models, we included the widely adopted technique known as ten-fold cross-validation. This technique involved the systematic division of the training dataset into ten subsets, each of which was subsequently utilized for model training and evaluation in a carefully orchestrated manner. Through this iterative process, the models were trained on nine subsets while being meticulously evaluated on the remaining subset. By aggregating the results across these iterations, a comprehensive and robust estimation of the models’ predictive capabilities was derived.

Multiple measures were utilized to analyze each model’s performance, Accuracy,

95% Accuracy Confidence Interval (CI), Kappa, Sensitivity, and Specificity. The performance measures for the four ML methods are reported in **Table 4**.

**Table 4:** Performance measures of four ML models.

	Accuracy	95% Accuracy CI	Kappa	Sensitivity	Specificity	AUC
RF	0.8389	(0.8002,0.8726)	0.6776	0.8419	0.8357	0.900
Bagged CART	0.8152	(0.7748,0.8511)	0.6306	0.7907	0.8406	0.892
XGBoost	0.7133	(0.6675, 0.756)	0.426	0.7349	0.6908	0.783
NN	0.6635	(0.6162, 0.7085)	0.3275	0.6419	0.6860	0.741

The Accuracy evaluation computes the models’ ability to detect instances related to the diagnosis of breast cancer. To achieve this, we compute the proportion of accurately classified instances to all occurrences, accounting for True Positive (TP), True Negative (TN), False Positive (FP), and False Negative (FN). Where True Positive (TP) is the number of correctly predicted positive instances, True Negative (TN) is the number of correctly predicted negative instances, False Positive (FP) is the number of incorrectly predicted positive instances, and False Negative (FN) is the number of incorrectly predicted negative instances.

Formula for calculating accuracy is as follows:


$$Accuracy=\frac{\left(True\;Positive+True\;Negative\right)}{\left(True\;Positive+False\;Positive\;+True\;Negative+False\;Negative\right)}$$

The RF and Bagged CART models achieved the highest accuracy scores of 0.8389 and 0.8152, respectively. The XGBoosting and NN models had relatively lower accuracy scores, obtaining 0.7133 and 0.6635, respectively.

The 95% Accuracy CI for the RF, Bagged CART, XGBoost, and NN models are (0.8002, 0.8726), (0.7748, 0.8511), (0.6675, 0.756), and (0.6162, 0.7085), respectively. The overlapping intervals suggest that there may not be statistically significant differences in the performance between the models. However, further analysis and consideration of other performance measures are necessary to make more robust conclusions about the model’s performance.

Kappa, a measure of inter-rater agreement in the diagnosis of breast cancer, the RF and Bagged CART models exhibited higher Kappa values of 0.6776 and 0.6306, respectively, indicating a substantial level of agreement beyond chance. Following widely accepted interpretation guidelines (33), these values are considered substantial. Conversely, the XGBoost and NN models displayed lower Kappa values of 0.426 and 0.3275, respectively, suggesting a moderate and slight level of agreement, respectively, which is comparatively lower for these models.

Sensitivity, which measures the model’s ability to correctly identify individuals with breast cancer, emphasizing the significance of true positive predictions, was determined. The RF model exhibited the highest sensitivity (0.8419), indicating its effectiveness in identifying true positive cases. Specificity, on the other hand, measures the model’s ability to correctly identify actual negative instances. The Bagged CART model had the highest specificity (0.8406), indicating its proficiency in identifying true negative cases.

The RF and Bagged CART models demonstrated superior performance in terms of accuracy, Kappa, sensitivity, and specificity compared to the XGBoost and NN models. However, the choice of the best model may ultimately depend on the specific application and the relative importance of sensitivity and specificity in the given context.

**Figure 2:**
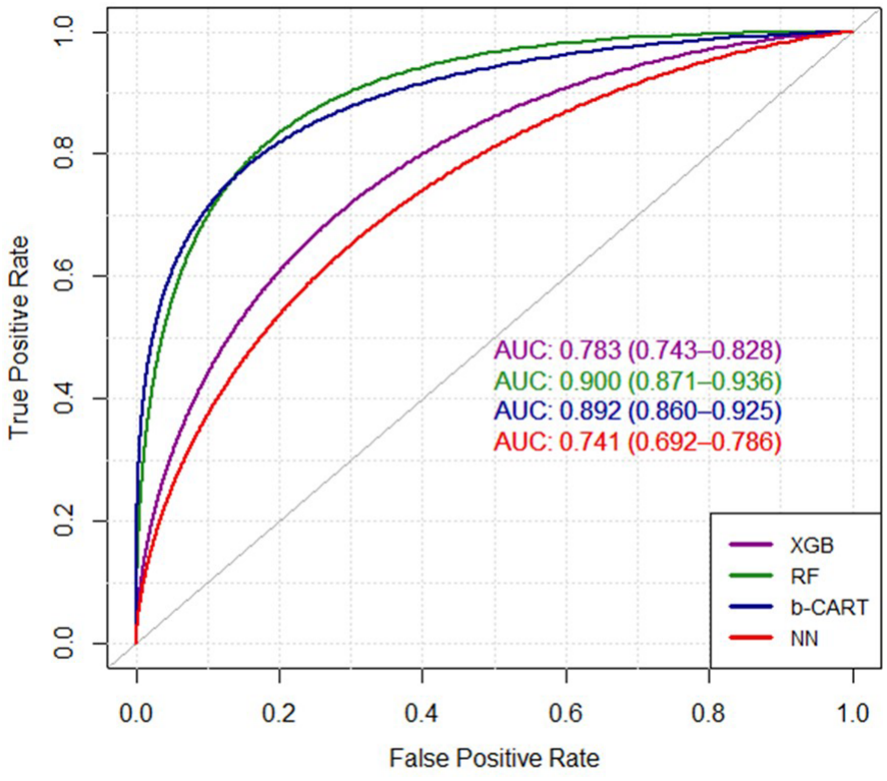
The ROC curves of four ML algorithms. XGB, extreme gradient boosting tree; RF, random forest; b-CART, bagged CART; NN, neural networks.

Finally, the evaluation of ROC/AUC revolves around the discriminating ability of the models in diagnosing breast cancer. The Receiver Operating Characteristic (ROC) curve serves as a valuable visual tool to evaluate the classification models’ performance, depicting the balance between correctly identifying positive cases and incorrectly classifying negative cases. The Area Under the Curve (AUC) metric provides a comprehensive measure of the models’ discriminative ability. **Figure 2** represents the ROC curves of the four ML models. In the context of the presented results, it is noteworthy that the RF model showcased the highest AUC value of 0.900, indicating its remarkable proficiency in effectively distinguishing between the classes. Following closely, the Bagged CART model demonstrated a commendable AUC of 0.892. In contrast, the XGBoost model exhibited a relatively lower AUC of 0.783, while the NN model displayed the lowest AUC of 0.741. These findings substantiate the superiority of the RF model in accurately classifying the data, thereby establishing its significance and prominence within the analytical assessment.

### 4.3 Ensemble models

Ensemble methodologies encompass learning strategies that aim to create a robust and improved predictive model through the integration of multiple learning algorithms (34). The integration of weak learners in ensemble learning techniques can be achieved through two approaches: homogeneous and heterogeneous ensembles (35). Weak learners, recognized as base models within the realm of ML, represent algorithms that outperform random guesses with a noticeable yet moderate degree of effectiveness. Ensemble learning reveals the principle of homogeneity, which is embodied through the well-established methodologies of bagging and boosting. These influential techniques are highly regarded for their capability to aggregate multiple instances of similar weak learners, resulting in homogeneous ensembles that possess enhanced predictive capabilities. Conversely, heterogeneous ensembles adopt a distinct and advanced strategy known as stacking, which involves integrating diverse weak learners. Distinguished by its heterogeneity, stacking augments the ensemble’s predictive capabilities by harnessing a synergistic interplay of complementary algorithms (36).

In this study, the application of stacking ensemble learning for predicting the intended outcome was undertaken, followed by a comparison of the results with the best-performing machine learning (ML) algorithm. In this research, the stacked generalization, commonly referred to as stacking, algorithm was employed. Initially, a variety of learning algorithms were utilized to generate predictions, and subsequently, the outcomes were integrated through the application of a combiner algorithm, which represents an additional technique within the field of ML. This integration process allowed for a comprehensive assessment of the predictive performance.

Ensemble methods aim to enhance model performance by combining diverse base models, characterized by low correlations among them. In the evaluation of the association between these models, the correlation coefficients span from -1 to 1. A value of -1 signifies a perfect negative correlation, while a value of 1 indicates a perfect positive correlation. Nevertheless, within the framework of ensemble methods, the ideal correlation value is 0, signifying an absence of any relationship between the models. **Table 5** displays the correlation coefficients among the base models used in ensemble learning.

**Table 5:** Base models correlation.

	XGBoost	NN	RF	Bagged CART
**XGBoost**	1.00	0.31	0.46	0.19
**NN**	0.31	1.00	0.53	0.18
**RF**	0.46	0.53	1.00	0.60
**Bagged CART**	0.19	0.18	0.60	1.00

The low correlation among base models is crucial as it indicates that the models provide independent and distinct perspectives which means they make different types of errors or predictions. This diversity allows the ensemble model to capture a wider range of patterns and insights, ultimately improving prediction accuracy. In other words, this diversity is beneficial because when combined, the ensemble can compensate for individual model weaknesses and exploit the strengths of different models. By considering various perspectives and incorporating different types of information, the ensemble can produce more accurate and robust predictions than any individual model alone. Conversely, high correlations between base models can introduce redundancy and limit the ensemble’s ability to incorporate diverse insights. By leveraging a diverse set of base models with low correlations, ensemble methods offer a robust and accurate approach to prediction and generalization in ML. The collective wisdom of these models, integrated using a combiner algorithm, allows the ensemble model to harness the strengths of each base model and mitigate potential biases, leading to enhanced predictive capabilities and generalization performance.

Ensemble models, such as those incorporating the Generalized Linear Model and Boosted Classification Trees, provide a framework for combining the predictive abilities of multiple models, thereby mitigating individual model weaknesses, and capitalizing on their strengths. By blending diverse perspectives and capturing a wider range of patterns, ensemble models can potentially achieve higher accuracy and agreement than any individual model alone.

**Table 6** presents the performance metrics of the best-performing previous model, RF, in comparison to two ensemble models: Generalized Linear Model and Boosted Classification Trees. These metrics provide insights into the models’ predictive capabilities and agreement with actual outcomes.

**Table 6:** Performance comparison of RF model with other ensemble models.

	Accuracy	Kappa
**RF**	83.89%	67.76%
**Generalized Linear Model**	83.00%	66.00%
**Boosted Classification Trees**	82.56%	65.12%

Regarding Accuracy and Kappa, the RF model achieved an accuracy of 83.89% and a Kappa coefficient of 67.76%. These results indicate that the RF model yielded correct predictions with an accuracy of 83.89% and demonstrated substantial agreement beyond what would be expected by chance. The Generalized Linear Model, when employed within the ensemble, achieved slightly lower accuracy and Kappa coefficient of 83.00% and 66.00%, respectively. Similarly, the Boosted Classification Trees, when incorporated in the ensemble, achieved a lower accuracy of 82.56% and lower Kappa coefficient of 0.65.

The RF model is known for its ability to handle complex relationships and interactions between variables. It combines multiple decision trees and aggregates their predictions to make accurate predictions. The results of **Table 6** indicate that the RF model outperformed both the Generalized Linear Model and the Boosted Classification Trees in terms of accuracy and agreement. However, it is essential to acknowledge that ensemble models have their own merits, offering the potential for improved performance by incorporating diverse models so they can offer complementary insights and diversify predictions, potentially enhancing performance in specific contexts. Choosing the most suitable model or ensemble approach depends on the specific characteristics of the data and the objectives of the analysis.

## 5 Discussion

BC remains a significant public health concern globally, and understanding the risk factors associated with the disease is crucial for effective prevention and intervention strategies. This study aimed to investigate BC risk factors among women in Iran’s Fars province, contributing valuable insights to the understanding of disease etiology and informing targeted approaches to BC prevention and management. The rigorous methodology employed in this study, including data collection from a representative sample size and the use of four ML algorithms, ensures the reliability and robustness of the findings.

The analysis of variable importance across the ML models revealed variations in the rankings of risk factors, which underscores the inherent complexity and heterogeneity of BC etiology. However, in line with the existing body of literature certain variables including chest X-ray history, deliberate weight loss, and abortion history consistently emerged as influential across all four algorithms, suggesting their potential significance as BC risk factors (37–40). Additionally, variables such as post-menopausal status, second-hand smoking, OCP use, and education (high school) were consistently identified as significant risk factors by the majority of the ML models echoing earlier investigations (8, 41–44).

In accordance with prior research findings, this study also indicates the factors that do not display significant involvement in the risk assessment of BC including occupation, physical activity, BMI, age at first delivery, breastfeeding, history of miscarriage, menarche age, regular menstruation, and menopausal status (19, 45–51).

By combining the power of ML algorithms with comprehensive risk factor assessment, significant strides can be made in mitigating the burden of BC among women in both Iran and around the globe. The ML models showcase robust performance metrics. Notably, RF and Bagged CART stand out with their higher accuracy and Kappa values, reinforcing their potential for accurate BC risk prediction. However, the reasonable sensitivity and specificity values exhibited by XGBoost and NN highlight their ability to identify both true positive and true negative cases. With all ML models exhibiting good discriminatory power, these findings emphasize the effectiveness of ML algorithms in assessing BC risk.

This study significantly contributes to the expanding body of research focused on identifying predictors of BC risk. The findings underscore the importance of employing multiple ML models to enhance the accuracy of BC risk prediction. Furthermore, the study emphasizes the necessity of considering a wide array of risk factors during model development. By incorporating these approaches, the accuracy and effectiveness of risk prediction can be heightened, ultimately alleviating the burden of BC among women in Iran. Continued research advancements are crucial to further deepen our understanding and develop targeted interventions for BC prevention and management. Also, it is crucial to recognize that the fight against BC is an ongoing battle. Continued research advancements are vital for deepening our understanding of the complex interplay between risk factors and BC etiology. Moreover, these findings should be complemented with translational efforts to ensure that evidence-based strategies are effectively disseminated and implemented in clinical practice and public health policies.

By integrating the insights from this study into comprehensive risk prediction models, public health practitioners can enhance their ability to identify individuals at high risk of developing BC, enabling timely interventions and personalized prevention strategies.

This study is a significant step toward improving breast cancer prevention, early detection, and management. Continued research advancements and collaborative efforts are crucial to further deepen our understanding, develop targeted interventions, and ultimately reduce the burden of breast cancer on women’s health worldwide following interventions on the (modifiable) risk actors.

Although our study has several strengths, including the application of different methods, considering a vast variety of potential confounders and risk factors, and a large sample size, we would like to bring some limitations to the table. We collected data from a reference hospital in the southern part of the country but did not include data from other geographic areas of the country. Hence, in future research, it might be significant to consider a representative sample of different parts of the country to find any discrepancies between different ethnic groups. To ensure that ethnicity or other socio-demographic factors did not affect our results, we adjusted the results for potential confounders in the southern part of the country. Also, recruiting participants who visited the biggest referral center in the southern part of Iran makes the results generalizable to the city’s population.

Another limitation is recall bias from a case-control study, which is a common bias in this study design. To address this limitation, we could use some objective variables that participants can remember. Another potential confounding factor is alcohol consumption, which is not legal in our country, making it difficult to determine its true impact. However, we expect minimal influence on our results from alcohol consumption, as it is not prevalent in our country.

## 6 Conclusion

BC is a complex disease with many different risk factors that influence it, necessitating a thorough analysis. This study underscores the application of ML models to identify significant predictors of BC risk, thereby enhancing risk prediction accuracy. Within the context of the study population, this research highlights the pivotal role of demographic, medical history, and lifestyle factors in evaluating BC risk among women. The study’s outcomes indicated that a history of chest X-rays emerged as a noteworthy risk factor for BC. Furthermore, the presence of a family history of BC, smoking habits, and OCPs usage were identified as substantial predictors. These findings suggest that interventions targeting smoking cessation and promoting BC screening among women with a familial BC history could yield effective outcomes in reducing BC incidence.

Moreover, this research provides valuable insights into the intricate interplay of metabolic and hormonal factors contributing to BC development. The identification of deliberate weight loss, abortion history, and post-menopausal status as significant predictors underscores the significance of considering multiple factors when assessing BC risk. By expanding the existing knowledge base on BC risk factors, this study emphasizes the utilization of advanced ML techniques to elucidate complex interactions among various predictors. Subsequent studies can leverage these findings to develop more precise and efficacious BC risk prediction models, empowering clinicians, and patients to make informed decisions regarding BC prevention and management.

## Data availability statement

The data analyzed in this study is subject to the following licenses/restrictions: The dataset was obtain from a healthcare center in Shiraz, Iran. Being obtained from different geographic can improve validity of the dataset. Requests to access these datasets should be directed to dianati.epid@gmail.com.

## Ethics statement

Ethical approval was not required for the study involving humans in accordance with the local legislation and institutional requirements. Written informed consent to participate in this study was not required from the participants or the participants’ legal guardians/next of kin in accordance with the national legislation and the institutional requirements.

## Author contributions

MD-N: Data curation, Software, Supervision, Writing – review & editing, Conceptualization, Formal analysis, Investigation, Methodology, Project administration, Resources, Validation. KS: Data curation, Supervision, Writing – review & editing, Conceptualization, Formal analysis, Investigation, Methodology, Project administration, Resources, Validation. RM: Formal analysis, Investigation, Methodology, Project administration, Resources, Validation, Writing – review & editing. SS: Investigation, Methodology, Software, Validation, Writing – review & editing. MF: Data curation, Supervision, Writing – review & editing, Conceptualization, Formal analysis, Investigation, Methodology, Project administration, Resources, Validation. KH: Data curation, Writing – review & editing. BJ-S: Methodology, Validation, Resources, Writing – review & editing. TC: Data curation, Software, Supervision, Writing – review & editing, Conceptualization, Formal analysis, Investigation, Methodology, Project administration, Resources, Validation. SD: Data curation, Software, Supervision, Writing – review & editing, Conceptualization, Formal analysis, Investigation, Methodology, Project administration, Resources, Validation.
